# Financial toxicity and its association with quality of life and psychological distress among patients with gynecologic cancers receiving radiotherapy in China: a multicenter cross-sectional study

**DOI:** 10.3389/fpubh.2026.1782189

**Published:** 2026-04-13

**Authors:** Jianjun Zhang, Jing Zhao, Zhilan Bai, Keqi Chen, Na Li, Jingwen He, Bi Jiang, Congwei Dai, Yan Zuo

**Affiliations:** 1 Department of Gynecology and Obstetrics, West China Second University Hospital, Sichuan University/Key Laboratory of Birth Defects and Related Diseases of Women and Children (Sichuan University), Ministry of Education, Chengdu, China; 2 Department of Oncology, Hebei General Hospital, Shijiazhuang, China; 3 Department of Gynecology and Obstetrics Nursing, West China Second University Hospital, Sichuan University / West China School of Nursing, Sichuan University / Key Laboratory of Birth Defects and Related Disease of Women and Children (Sichuan University), Ministry of Education, Chengdu, China; 4 Department of Gastroenterology, West China Fourth Hospital, Sichuan University, Chengdu, China; 5 Gynecology Department, Hebei General Hospital, Shijiazhuang, China

**Keywords:** China, cost, financial toxicity, gynecologic cancer, psychological distress, quality of life, radiotherapy

## Abstract

**Background:**

Financial toxicity is increasingly recognized as a consequential dimension of cancer care, yet multicenter evidence in China remains limited for patients with gynecologic cancers undergoing radiotherapy, a treatment pathway that often entails repeated visits and substantial non-medical costs. This study estimated the prevalence and severity of financial toxicity and examined its association with quality of life and psychological distress.

**Methods:**

A multicenter cross-sectional survey was conducted in three tertiary hospitals in China, led by West China Second University Hospital, Sichuan University. Adult patients with gynecologic cancers receiving external beam radiotherapy and or brachytherapy with definitive, adjuvant, or curative-intent salvage or consolidation intent were consecutively recruited. Financial toxicity was assessed using the COmprehensive Score for financial Toxicity (COST, 0 to 44; lower scores indicate worse toxicity). Quality of life was measured using the EORTC QLQ-C30, and psychological distress using the Distress Thermometer (DT, 0 to 10; clinically significant distress defined as DT ≥ 4). Multivariable regression models included hospital fixed effects to account for measured differences across centers and adjusted for sociodemographic, access-burden, and clinical covariates.

**Results:**

Among 1,533 returned questionnaires, 1,303 were valid and analyzed (85.0%). Mean COST score was 21.6 (SD 7.4); 17.5% had severe financial toxicity (COST ≤ 14), 48.0% moderate (15 to 24), and 34.5% mild (≥25). Mean QLQ-C30 global health status was 61.3 (SD 14.2). Mean DT score was 4.3 (SD 2.1), and 65.2% met criteria for clinically significant distress. In adjusted analyses, each 5-point decrease in COST was associated with lower global health status (β −4.17, 95% CI −4.62 to −3.72), lower emotional functioning (β −4.68, 95% CI −5.20 to −4.16), higher fatigue (β 4.47, 95% CI 3.93 to 5.01), and higher DT score (β 0.32, 95% CI 0.23 to 0.40), all *p* < 0.001. Each 5-point decrease in COST was associated with higher odds of clinically significant distress (OR 1.34, 95% CI 1.22 to 1.47; *p* < 0.001).

**Conclusion:**

Financial toxicity was common among gynecologic radiotherapy patients in China and was independently associated with poorer quality of life and higher psychological distress. Integrating financial toxicity screening with supportive care pathways during radiotherapy may help identify high-risk patients and guide targeted assistance.

## Introduction

Gynecologic cancers remain a major contributor to cancer morbidity among women in China and include cervical, endometrial, ovarian, vaginal, and vulvar malignancies (1). Radiotherapy is integral across this disease spectrum (2). It is central to curative management for many patients with cervical and vaginal cancers, is commonly used as adjuvant pelvic radiotherapy and or vaginal brachytherapy for endometrial cancer, and is also used for selected ovarian and other gynecologic cancers in postoperative, consolidative, or curative-intent salvage settings such as localized or nodal disease control in tertiary practice (3). As survival improves and treatment pathways become more complex, the burden of care increasingly extends beyond tumor control to include patient-centered outcomes such as health-related quality of life and psychological wellbeing (4). In parallel, the financial consequences of cancer care have become a visible and consequential dimension of cancer survivorship and equity (5, 6).

The concept of “financial toxicity” was introduced to describe the harmful financial burden and financial distress that can arise from cancer and its treatment, analogous in importance to physical and psychological toxicities (7). Financial toxicity is typically framed as a multidimensional construct that includes objective financial burden such as out-of-pocket payments, direct non-medical costs, and lost income, together with subjective financial distress and cost-coping behaviors such as borrowing, reducing essential consumption, delaying care, or non-adherence (6, 8). The policy relevance is clear: financial hardship can limit access to appropriate care, intensify stress, and contribute to disparities in outcomes that are not explained by clinical factors alone (4, 5).

To translate this policy concern into clinical and health-system action, financial toxicity must also be measured in a standardized patient-reported way, so that burden can be identified consistently, compared across populations, and linked to potentially modifiable care and financing factors (9). The COmprehensive Score for financial Toxicity (COST) instrument was developed as a clinically relevant measure of perceived financial distress in cancer care and has become one of the most widely used tools in the field (2). COST has been used across diverse oncology populations and has motivated translation and psychometric validation work in multiple settings, including Chinese-language versions (5, 10). A growing literature now treats financial toxicity as an actionable domain for screening and intervention, aligning with broader efforts in supportive care in oncology to identify modifiable threats to treatment completion, symptom management, and mental health (3, 4).

International evidence consistently indicates that financial toxicity is common and associated with worse patient outcomes (8). Systematic reviews and meta-analyses have reported substantial prevalence of both objective financial hardship and subjective financial toxicity across income settings, with heterogeneity driven by insurance design, household resources, and treatment modality (11). Evidence also links financial toxicity to reduced health-related quality of life, reinforcing that economic strain can translate into impaired functioning and symptom burden (9, 12). Parallel work highlights associations between financial hardship and mental health outcomes including anxiety and depression, supporting the plausibility of a pathway from cost pressure to psychological distress (4, 13). Although the directionality and causality can be difficult to establish in observational designs, the overall pattern across studies suggests financial toxicity is not merely a byproduct of disease severity but an independent correlate of wellbeing with potential downstream effects (1).

China provides a particularly important context for studying financial toxicity because several structural features of the health system may shape both its magnitude and distribution. Cancer care is delivered within a mixed insurance environment that includes differences in reimbursement generosity, deductibles, covered services, and portability across schemes, including the Urban Employee Basic Medical Insurance (UEBMI) and Urban-Rural Resident Basic Medical Insurance (URRBMI) systems, while out-of-pocket payment remains substantial for many households, especially when treatment requires care outside the patient's home region (10). In addition, direct non-medical costs such as travel, lodging, meals, and caregiver time are often less well protected than formal medical charges, and urban-rural differences in access to tertiary oncology services may further concentrate burden among already vulnerable families (5, 8). For this reason, evidence from Western settings cannot simply be assumed to generalize to China: the configuration of insurance protection, referral patterns, and non-medical treatment burden differs in ways that may alter both the severity and the determinants of financial toxicity. Existing studies from China show that financial toxicity is common, has been measured with heterogeneous tools, and is associated with adverse health-related outcomes, while also highlighting the need for more standardized measurement and population-specific evidence (1, 9, 10).

Radiotherapy is a particularly relevant treatment context for financial toxicity in gynecologic cancers, including in the Chinese tertiary-care setting examined here, because it is time-intensive and logistically demanding across multiple disease sites and treatment indications (2). Radiotherapy commonly requires repeated visits over several weeks, and the prolonged, fractionated treatment course can magnify travel-related financial and time costs (3). This burden may be especially pronounced when patients receive care at tertiary referral centers and must travel across regions for definitive treatment, adjuvant therapy, or curative-intent salvage or consolidation (5). Transportation, lodging, meals, caregiver time, and foregone income can therefore create a substantial non-medical cost burden that is not fully offset by reimbursement for medical services alone (6). In many cases, radiotherapy is also combined with brachytherapy and or concurrent chemotherapy, increasing care complexity and potentially intensifying both financial strain and symptom burden (14). Because radiotherapy may intensify both economic and practical treatment burden, its consequences may be especially visible in patient-reported outcomes that reflect daily functioning and emotional wellbeing.

In particular, the consequences are likely to be salient for health-related quality of life and psychological distress, outcomes that matter to patients and to health systems focused on value-based care (4). Standardized instruments support rigorous evaluation in China. The EORTC QLQ-C30 has been translated and validated in Chinese populations, providing a well-established framework for global health status, functioning, and symptoms (10). Psychological distress screening using the NCCN Distress Thermometer has also been validated in Chinese cancer patients, supporting its use for identifying clinically meaningful distress burden (5). Recent studies continue to highlight the intersection of financial toxicity and psychological outcomes in cancer populations, including work in China that has examined financial toxicity alongside psychological distress (1). Yet, despite expanding research, multicenter evidence that focuses on patients actively receiving radiotherapy for gynecologic cancers in China remains limited, particularly evidence that evaluates financial toxicity in relation to both quality of life domains and distress while accounting for socioeconomic conditions, access burdens, and clinical characteristics (2).

To address this gap, the present multicenter cross-sectional study surveyed patients with gynecologic cancers receiving radiotherapy at three tertiary hospitals in China. The primary aims were to quantify the prevalence and severity of financial toxicity and to examine its association with quality of life and psychological distress using validated patient-reported outcome measures. We hypothesized that worse financial toxicity would be associated with lower global health status and functioning, higher symptom burden, higher distress, and greater odds of clinically significant distress after adjustment for sociodemographic factors, access burdens such as travel and lodging needs, and clinical variables related to cancer type, stage, treatment modality, and performance status.

## Methods

### Study design and setting

A multicenter cross-sectional survey was conducted among patients with gynecologic cancers receiving radiotherapy in China. Three tertiary hospitals participated, with West China Second University Hospital, Sichuan University serving as the principal investigator (PI, Center 1) institution and coordinating center. The other two centers included West China Fourth Hospital, Sichuan University (Center 2) and Hebei General Hospital (Center 3). Hospitals were selected purposively because they were tertiary centers with established gynecologic oncology and radiotherapy services, sufficient patient volume for consecutive recruitment, and operational capacity to implement a unified multicenter protocol. The inclusion of centers from different regions was intended to improve heterogeneity in referral patterns and patient access burden. All participating centers used a unified study protocol, standardized questionnaire administration procedures, and harmonized variable definitions. Eligible patients were recruited consecutively between August 3, 2025 and October 27, 2025 in outpatient radiotherapy units and inpatient wards.

### Participants

Participants were eligible if they met all of the following criteria: age 18 years or older; pathologically confirmed gynecologic malignancy including cervical, endometrial, ovarian, vaginal, vulvar, or other gynecologic cancers; currently receiving external beam radiotherapy and or brachytherapy delivered with definitive or adjuvant intent, or delivered as curative-intent salvage or consolidation for localized or regional disease control as determined by the treating team; ability to communicate in Chinese and complete questionnaires independently or with minimal neutral assistance; and provision of written informed consent. Exclusion criteria included severe cognitive impairment or psychiatric instability precluding valid questionnaire completion as judged by treating clinicians, critical illness requiring intensive care, exclusive palliative radiotherapy without curative or adjuvant intent, and repeat participation. Patients receiving exclusive palliative radiotherapy were excluded to preserve clinical comparability within the analytic cohort, because palliative treatment settings often differ substantially from curative or adjuvant settings with respect to prognosis, symptom burden, care trajectories, and supportive needs. This was intended to reduce clinical heterogeneity in analyses of financial toxicity, quality of life, and distress, although it also limits generalizability to patients receiving palliative radiotherapy.

### Sampling and recruitment procedures

At each hospital, trained research staff screened radiotherapy appointment lists and inpatient rosters to identify potentially eligible patients. A non-probabilistic consecutive sampling strategy was used, whereby all eligible patients encountered during the recruitment period at participating outpatient radiotherapy units and inpatient wards were invited to participate. After confirming eligibility, research staff introduced the study, obtained written informed consent, and provided the questionnaire in a quiet area before or after radiotherapy. Questionnaires were typically completed within 15–25 min. For participants with limited literacy or visual impairment, staff read items verbatim and recorded responses without interpretation. Participation did not influence clinical decision-making.

### Measures and operational definitions

#### Financial toxicity

Financial toxicity was assessed using the Comprehensive Score for Financial Toxicity (COST), an 11-item patient-reported instrument designed to capture perceived financial distress related to cancer care. Items are rated on 5-point Likert-type response options and summed according to standard scoring procedures; several items are reverse scored before aggregation so that the total score ranges from 0 to 44, with lower scores indicating worse financial toxicity. For descriptive analyses, COST severity categories were prespecified as severe ( ≤ 14), moderate (15-24), and mild (≥25) (15). Internal consistency of the COST scale in the present cohort was evaluated using Cronbach's α and McDonald's ω.

#### Quality of life

Health-related quality of life was assessed using the European Organization for Research and Treatment of Cancer Quality of Life Questionnaire Core 30 (EORTC QLQ-C30, version 3.0). Standard scoring procedures were applied to transform raw scores to 0–100 scales. Higher scores represent better functioning for functional scales and global health status, whereas higher scores represent worse symptom burden for symptom scales (16). Internal consistency of the multi-item QLQ-C30 scales used in the present analyses was evaluated in the study cohort using Cronbach's α.

#### Psychological distress

Psychological distress was measured using the Distress Thermometer (DT), ranging from 0 to 10. Clinically significant distress was defined a priori as DT ≥ 4 for binary outcome analyses, and DT was also analyzed as a continuous outcome (17).

#### Covariates

Sociodemographic variables included age, residence (urban/rural), education, employment status, household income category, insurance type, travel distance to treating hospital, and indicators of care burden (cross-region care, lodging needs during radiotherapy). Clinical variables extracted from medical records included cancer type, stage, treatment intent (definitive/adjuvant), radiotherapy modality (external beam radiotherapy alone, brachytherapy alone, combined modality), concurrent chemotherapy, ECOG performance status, comorbidity status, and time since diagnosis.

### Data management and quality control

All hospitals followed a centralized operations manual and completed standardized staff training on screening, consent, questionnaire administration, and data handling. Questionnaires were checked for completeness at the time of collection when feasible. Data were entered into a secure electronic database with prespecified range and logic checks. Participants were identified using coded study IDs; identifying information was stored separately with restricted access.

### Statistical analysis

Continuous variables were summarized using mean (SD) when distributions were approximately symmetric and median (IQR) when skewness was evident; categorical variables were summarized using counts and percentages. Distributional assumptions for continuous variables were assessed using histograms, Q-Q plots, and Shapiro-Wilk tests, and homogeneity of variance for ANOVA comparisons was evaluated using Levene's test. Differences across COST severity categories were evaluated using chi-square tests for categorical variables, one-way ANOVA for continuous variables judged to meet approximate parametric assumptions, and Kruskal-Wallis tests otherwise. Associations between COST and patient-reported outcomes were first examined using Pearson correlations when approximate linearity and distributional assumptions were acceptable; Spearman rank correlations were also examined as a sensitivity analysis for skewed or bounded measures.

Multivariable associations between COST and outcomes were evaluated using regression models accounting for the multicenter structure. Given the small number of participating hospitals, hospital-level fixed effects were included to account for measured differences across centers. For quality-of-life outcomes (QLQ-C30 scales) and continuous distress (DT score), linear regression models were used; for clinically significant distress (DT ≥ 4), logistic regression was used. Model diagnostics for linear regression included assessment of residual normality, linearity, homoscedasticity, and influential observations using residual plots and Q-Q plots; heteroscedasticity was further evaluated using the Breusch-Pagan test. Multicollinearity among covariates was assessed using variance inflation factors. COST was modeled continuously, and the primary effect was expressed per 5-point decrease in COST to improve interpretability. All models were adjusted for prespecified sociodemographic and clinical covariates.

### Ethical considerations

The study protocol was ethically approved by the Ethics Committee of West China Second University Hospital, Sichuan University (Approval number 2025-134). All participants provided written informed consent prior to enrollment. Confidentiality was protected via coded identifiers, restricted access to linkage files, and secure data storage.

## Results

### Participant flow and data completeness

Across the three tertiary hospitals, 1,533 questionnaires were returned. After data cleaning, 1,303 questionnaires were judged valid and included in the analysis (85.0%). A total of 230 questionnaires were excluded due to substantial missingness in at least one key patient-reported component (COST, Distress Thermometer, or QLQ-C30 core scoring items) and or incomplete core demographic or clinical fields required for adjusted models.

### Participant characteristics

Among 1,303 participants, 590 (45.3%) were recruited at Center 1, 382 (29.3%) at Center 2, and 331 (25.4%) at Center 3. Mean age was 52.3 years (SD 10.1). Urban residence accounted for 54.7% of the cohort. Urban-Rural Resident Basic Medical Insurance (URRBMI) was the most common insurance type (55.4%), followed by Urban Employee Basic Medical Insurance (UEBMI) (33.5%). Cross-region care was reported by 27.6%, and 32.1% reported lodging needs during radiotherapy. Cervical cancer was the most common diagnosis (56.5%), and stage III-IV disease accounted for 61.3%. Among participants classified as ovarian cancer, radiotherapy was administered under definitive, adjuvant, or curative-intent salvage or consolidation indications, reflecting tertiary practice patterns such as involved-site or regional nodal irradiation and postoperative or localized disease control.

Financial toxicity was frequent. The mean COST score was 21.6 (SD 7.4). Severe financial toxicity (COST ≤ 14) occurred in 228 participants (17.5%), moderate toxicity (15-24) in 625 (48.0%), and mild toxicity (≥25) in 450 (34.5%). Several socioeconomic burden markers differed across COST categories, including income distribution, distance to hospital, and out-of-pocket spending (Table 1).

**Table 1:** Participant characteristics overall and by financial toxicity severity (COST categories).

Characteristic	Overall	Severe ( ≤ 14)	Moderate (15-24)	Mild (≥25)	*P* value^*^
Hospital					< 0.001
Center 1	590 (45.3)	148 (64.9)	295 (47.2)	147 (32.7)	
Center 2	382 (29.3)	46 (20.2)	183 (29.3)	153 (34.0)	
Center 3	331 (25.4)	34 (14.9)	147 (23.5)	150 (33.3)	
Age, years	52.3 (10.1)	52.1 (10.0)	52.0 (10.0)	52.9 (10.3)	0.272
Residence					< 0.001
Rural	590 (45.3)	143 (62.7)	300 (48.0)	147 (32.7)	
Urban	713 (54.7)	85 (37.3)	325 (52.0)	303 (67.3)	
Education					< 0.001
≤ Middle school	586 (45.0)	139 (61.0)	298 (47.7)	149 (33.1)	
College	247 (19.0)	17 (7.5)	113 (18.1)	117 (26.0)	
High school/technical	470 (36.1)	72 (31.6)	214 (34.2)	184 (40.9)	
Employment					< 0.001
Employed	467 (35.8)	70 (30.7)	220 (35.2)	177 (39.3)	
Not employed/retired	836 (64.2)	158 (69.3)	405 (64.8)	273 (60.7)	
Household monthly income (RMB)					< 0.001
12,000–19,999	151 (11.6)	0 (0.0)	43 (6.9)	108 (24.0)	
3,000–4,999	282 (21.6)	76 (33.3)	153 (24.5)	53 (11.8)	
5,000–7,999	240 (18.4)	58 (25.4)	126 (20.2)	56 (12.4)	
8,000–11,999	308 (23.6)	69 (30.3)	182 (29.1)	57 (12.7)	
< 3,000	206 (15.8)	24 (10.5)	106 (17.0)	76 (16.9)	
≥20,000	116 (8.9)	1 (0.4)	15 (2.4)	100 (22.2)	
Insurance					< 0.001
Commercial	133 (10.2)	7 (3.1)	52 (8.3)	74 (16.4)	
Other/none	11 (0.8)	2 (0.9)	5 (0.8)	4 (0.9)	
UEBMI	437 (33.5)	45 (19.7)	186 (29.8)	206 (45.8)	
URRBMI	722 (55.4)	174 (76.3)	382 (61.1)	166 (36.9)	
Travel distance to treating hospital					< 0.001
100–299 km	336 (25.8)	54 (23.7)	166 (26.6)	116 (25.8)	
10–49 km	335 (25.7)	44 (19.3)	162 (25.9)	129 (28.7)	
50–99 km	264 (20.3)	30 (13.2)	113 (18.1)	121 (26.9)	
< 10 km	214 (16.4)	23 (10.1)	105 (16.8)	86 (19.1)	
≥300 km	154 (11.8)	77 (33.8)	79 (12.6)	2 (0.4)	
Cross-region care					0.003
Yes	360 (27.6)	79 (34.6)	168 (26.9)	113 (25.1)	
No	943 (72.4)	149 (65.4)	457 (73.1)	337 (74.9)	
Lodging needed during radiotherapy					< 0.001
Yes	418 (32.1)	118 (51.8)	207 (33.1)	93 (20.7)	
No	885 (67.9)	110 (48.2)	418 (66.9)	357 (79.3)	
Out-of-pocket medical spending in past 4 weeks, RMB	4,300 (3,200–6,900)	7,100 (5,600–9,000)	4,300 (3,200–6,100)	3,000 (2,200–4,300)	< 0.001
Out-of-pocket non-medical spending in past 4 weeks, RMB	1,800 (900–3,300)	3,000 (2,000–4,300)	1,900 (1,000–3,300)	1,200 (600–2,100)	< 0.001
Cancer type					< 0.001
Cervical	737 (56.5)	157 (68.9)	359 (57.4)	221 (49.1)	
Endometrial	234 (18.0)	36 (15.8)	118 (18.9)	80 (17.8)	
Ovarian	234 (18.0)	35 (15.4)	118 (18.9)	81 (18.0)	
Other	98 (7.5)	0 (0.0)	30 (4.8)	68 (15.1)	
Stage					< 0.001
I-II	473 (36.3)	57 (25.0)	216 (34.6)	200 (44.4)	
III-IV	799 (61.3)	165 (72.4)	395 (63.2)	239 (53.1)	
Unknown	31 (2.4)	6 (2.6)	14 (2.2)	11 (2.4)	
Treatment intent					< 0.001
Adjuvant	492 (37.8)	56 (24.6)	229 (36.6)	207 (46.0)	
Definitive	811 (62.2)	172 (75.4)	396 (63.4)	243 (54.0)	
Radiotherapy modality					< 0.001
Brachytherapy only	210 (16.1)	23 (10.1)	88 (14.1)	99 (22.0)	
Combined EBRT + brachytherapy	519 (39.8)	133 (58.3)	262 (41.9)	124 (27.6)	
EBRT only	574 (44.1)	72 (31.6)	275 (44.0)	227 (50.4)	
Concurrent chemotherapy					0.001
Yes	606 (46.5)	126 (55.3)	296 (47.4)	184 (40.9)	
No	697 (53.5)	102 (44.7)	329 (52.6)	266 (59.1)	
ECOG performance status					< 0.001
0	446 (34.2)	41 (18.0)	202 (32.3)	203 (45.1)	
1	616 (47.3)	118 (51.8)	311 (49.8)	187 (41.6)	
2	212 (16.3)	59 (25.9)	105 (16.8)	48 (10.7)	
3	29 (2.2)	10 (4.4)	7 (1.1)	12 (2.7)	

^*^p values: comparisons across COST severity groups using chi-square tests for categorical variables, one-way ANOVA for age, and Kruskal-Wallis tests for out-of-pocket spending.

### Quality of life and psychological distress by financial toxicity

Overall QLQ-C30 global health status was 61.3 (SD 14.2). A monotonic pattern was observed across COST severity strata, with progressively worse quality-of-life and distress outcomes at lower COST levels. Participants with severe financial toxicity reported worse global health status and functioning, and higher symptom burden. For instance, global health status was 55.7 (SD 12.8) in the severe group and 65.9 (SD 14.1) in the mild group. Fatigue decreased from 56.4 (SD 15.9) in the severe group to 34.9 (SD 15.5) in the mild group. The QLQ-C30 financial difficulties item showed the largest between-group separation, with a mean of 67.4 (SD 15.9) in the severe group and 39.7 (SD 16.7) in the mild group (Table 2).

**Table 2:** Financial toxicity, quality of life, and distress outcomes by COST categories.

Outcome	Overall	Severe ( ≤ 14)	Moderate (15-24)	Mild (≥25)	*p* value
COST total score (0–44, lower worse)	21.6 (7.4)	10.8 (3.3)	19.8 (2.8)	29.5 (3.9)	< 0.001
EORTC QLQ–C30					
Global health status	61.3 (14.2)	55.7 (12.8)	60.1 (13.8)	65.9 (14.1)	< 0.001
Physical functioning	56.1 (13.9)	49.8 (13.6)	54.6 (13.2)	61.2 (13.2)	< 0.001
Role functioning	55.8 (16.0)	49.9 (15.6)	54.5 (15.5)	60.6 (15.7)	< 0.001
Emotional functioning	61.5 (14.9)	54.8 (14.8)	60.1 (14.3)	66.8 (14.1)	< 0.001
Cognitive functioning	63.1 (12.4)	62.2 (13.2)	62.4 (11.9)	64.5 (12.5)	0.009
Social functioning	56.4 (16.0)	50.4 (14.8)	55.8 (15.8)	60.3 (15.8)	< 0.001
Fatigue (symptom)	44.0 (17.3)	56.4 (15.9)	46.0 (15.6)	34.9 (15.5)	< 0.001
Pain (symptom)	42.0 (14.7)	50.0 (13.0)	42.7 (14.1)	37.0 (14.4)	< 0.001
Financial difficulties (single item)	51.0 (18.5)	67.4 (15.9)	53.1 (15.1)	39.7 (16.7)	< 0.001
Distress Thermometer (0–10)	4.3 (2.1)	5.1 (1.9)	4.4 (2.0)	3.8 (2.0)	< 0.001
Clinically significant distress (DT ≥ 4)	849 (65.2)	177 (77.6)	424 (67.8)	248 (55.1)	< 0.001

Psychological distress was common. Mean DT score was 4.3 (SD 2.1), and 849 participants (65.2%) met the prespecified threshold for clinically significant distress (DT ≥ 4). Distress prevalence was highest in the severe financial toxicity group (77.6%) and lowest in the mild group (55.1%) (Table 2).

Internal consistency in the present cohort was acceptable to good. For COST, Cronbach's α was 0.86 and McDonald's ω was 0.87. For the QLQ-C30 multi-item scales included in the main analyses, Cronbach's α values were 0.82 for physical functioning, 0.84 for role functioning, 0.88 for emotional functioning, 0.73 for cognitive functioning, 0.79 for social functioning, 0.85 for fatigue, and 0.81 for pain.

### Bivariate associations between financial toxicity, quality of life, and distress

COST was positively correlated with QLQ-C30 global health status and functioning scales, and negatively correlated with symptom burden and distress. The strongest bivariate association was observed between COST and the QLQ-C30 financial difficulties item (*r* = −0.585, *p* < 0.001). COST was also negatively correlated with DT score (*r* = −0.236, *p* < 0.001) (Table 3). As a sensitivity analysis, Spearman rank correlations were examined in parallel with Pearson correlations. The overall pattern of results was unchanged, with lower COST scores consistently associated with poorer global health status and functioning, higher symptom burden, greater perceived financial difficulties, and higher distress. The strongest association remained that between COST and QLQ-C30 financial difficulties (Pearson *r* = −0.585; Spearman rho = −0.565; both *p* < 0.001).

**Table 3:** Pearson correlations between COST and patient-reported outcomes.

Outcome	*r*	*p*
EORTC QLQ-C30		
Global health status	0.293	< 0.001
Physical functioning	0.313	< 0.001
Emotional functioning	0.31	< 0.001
Fatigue	−0.478	< 0.001
Pain	−0.335	< 0.001
Financial difficulties	−0.585	< 0.001
Distress thermometer score	−0.236	< 0.001

### Multivariable associations of financial toxicity with quality of life and distress

In adjusted models incorporating hospital fixed effects and prespecified sociodemographic and clinical covariates, lower COST scores remained independently associated with poorer quality of life and higher distress. Each 5-point decrease in COST was associated with a 4.17-point decrease in QLQ-C30 global health status and a 4.68-point decrease in emotional functioning. Symptom burden increased with worsening financial toxicity, with a 4.47-point increase in fatigue per 5-point decrease in COST. Distress also increased, with a 0.32-point increase in DT score per 5-point decrease in COST (Table 4).

**Table 4:** Adjusted associations of financial toxicity (COST) with QoL and distress (linear models).

Outcome	β per 5-point decrease in COST	95% CI	*p*
QLQ-C30			
Global health status	−4.17	−4.62 to −3.72	< 0.001
Emotional functioning	−4.68	−5.20 to −4.16	< 0.001
Fatigue (symptom)	4.47	3.93 to 5.01	< 0.001
Distress thermometer score	0.32	0.23 to 0.40	< 0.001

In the adjusted logistic regression model for clinically significant distress (DT ≥ 4), lower COST scores remained independently associated with distress. Each 5-point decrease in COST was associated with 34% higher odds of clinically significant distress (OR 1.34, 95% CI 1.22 to 1.47; *p* < 0.001). Advanced stage (III-IV vs. I-II) and poorer performance status (ECOG ≥ 2 vs. 0) were also associated with higher odds of distress (Table 5).

**Table 5:** Adjusted associations with clinically significant distress (DT ≥ 4) (logistic model).

Predictor	OR	95% CI	*p*
COST (per 5-point decrease)	1.34	1.22 to 1.47	< 0.001
Age (per 10 years)	1.18	1.04 to 1.33	0.008
Household income category (per level)	0.99	0.90 to 1.10	0.882
Cross–region care (yes vs. no)	1.09	0.82 to 1.43	0.56
Lodging needed during radiotherapy (yes vs. no)	1.18	0.91 to 1.53	0.216
Concurrent chemotherapy (yes vs. no)	1	0.78 to 1.28	0.983
Any comorbidity (yes vs. no)	0.84	0.66 to 1.07	0.165
Residence (ref: Rural)			
Urban	0.8	0.63 to 1.03	0.08
Insurance (ref: URRBMI)			
UEBMI	0.87	0.67 to 1.13	0.303
Commercial	1.17	0.77 to 1.78	0.455
Other/none	1.23	0.26 to 5.81	0.795
Distance to hospital (ref:<10 km)			
10–49 km	1.13	0.80 to 1.62	0.487
50–99 km	1.06	0.70 to 1.59	0.791
100–299 km	0.96	0.65 to 1.41	0.834
≥300 km	1.43	0.89 to 2.30	0.135
Stage (ref: I–II)			
III–IV	1.33	1.03 to 1.72	0.029
Unknown	1.33	0.73 to 2.42	0.357
ECOG (ref: 0)			
1	1.2	0.91 to 1.60	0.204
≥2	2.56	1.78 to 3.69	< 0.001

Models include hospital fixed effects and adjust for age, residence, education, employment, household income, insurance, travel distance, cross-region care, lodging needs, cancer type, stage, treatment intent, radiotherapy modality, concurrent chemotherapy, ECOG, comorbidity status, and time since diagnosis. Regression diagnostics did not indicate major violations of linear-model assumptions. Residual plots and Q-Q plots were broadly acceptable, sampled Shapiro-Wilk tests of residuals were non-significant across fitted linear models, Breusch-Pagan tests did not suggest important heteroscedasticity, and multicollinearity was limited (maximum variance inflation factor = 5.07).

## Discussion

This multicenter survey among patients with gynecologic cancers receiving radiotherapy in China indicates that financial toxicity is common and clinically meaningful in magnitude, with a sizable fraction of participants meeting criteria for severe financial toxicity on COST. Financial toxicity showed a clear socioeconomic and access-burden gradient, with worse scores concentrated among lower-income participants, rural residents, and patients reporting longer travel distances, cross-region care, lodging needs, and higher out-of-pocket spending. Financial toxicity was also patterned by clinical intensity markers, including definitive treatment intent, combined-modality radiotherapy, concurrent chemotherapy, advanced stage, and poorer performance status. Across patient-reported outcomes, worsening financial toxicity was associated with lower QLQ-C30 global health status and functioning, higher symptom burden, and higher psychological distress. The association was strongest for the QLQ-C30 financial difficulty item, consistent with conceptual overlap between perceived financial strain and financial distress. Importantly, multivariable models that adjusted for sociodemographic, access-burden, and clinical variables retained statistically robust associations between worsening financial toxicity and both quality of life and distress, supporting financial toxicity as an independent correlate rather than a simple proxy for disease severity or social disadvantage.

The present findings should also be interpreted against a broader international literature, including evidence from Western and European settings. Studies using the COST instrument in diverse oncology populations have similarly reported that worse financial toxicity is associated with poorer quality of life and greater psychological burden, suggesting that the relationship observed here is not unique to China but part of a broader and reproducible pattern across health systems. Recent radiotherapy-focused and gynecologic oncology studies from Western settings have reported comparable directional associations between COST scores, quality of life, and distress-related outcomes, supporting the external interpretability of the present findings while underscoring differences in health-system context. At the same time, cross-country comparison should be made cautiously because absolute COST levels and the relative importance of specific drivers may vary according to insurance design, reimbursement policy, and the extent to which non-medical costs are protected. In this sense, the present results appear internationally concordant in direction while remaining context-specific in mechanism and policy relevance.

The prevalence pattern aligns with a broad international literature showing that financial toxicity affects a substantial proportion of people with cancer and varies across health systems, disease sites, and patient characteristics (8, 18). Recent systematic reviews and meta-analyses have characterized financial toxicity as a global burden and have highlighted consistent risk factors such as lower income, limited insurance protection, and treatment intensity (19). Work focusing on COST-specific evidence has also shown that lower COST scores associate with worse health-related quality of life, reinforcing the clinical relevance of the construct (20). This interpretation is also consistent with recent ovarian-cancer literature reporting substantial financial hardship among patients with advanced disease, reinforcing that gynecologic malignancies can carry marked economic burden across treatment settings and disease stages. Recent work has also proposed a COST cut-off of 17.5 to indicate high financial toxicity associated with low quality of life, which provides a useful point of comparison for interpreting clinically meaningful burden in the present cohort, even though our primary descriptive categories followed previously specified severity bands (21). In an additional descriptive analysis using this threshold, 29.5% of the present cohort met criteria for high financial toxicity. A China-focused comment has emphasized that financial toxicity remains an under-recognized dimension of cancer care despite expanding coverage, and has called for integration of measurement and mitigation into routine oncology practice (22). Empirical work in China has supported the feasibility and validity of COST, including multi-center validation efforts and subsequent work seeking practical cut-offs linked to adverse behaviors or outcomes (1, 5). The socioeconomic gradients observed here are therefore directionally consistent with the broader China evidence base, in which lower household resources and vulnerable social positions predict greater financial distress (23, 24).

Radiotherapy is a context in which financial toxicity can be amplified because the care pathway is prolonged and visit-intensive (2). A radiotherapy-focused cohort study in a universal health care setting found that financial toxicity can remain common even when formal coverage is extensive, suggesting that non-medical costs, time costs, and income disruption can sustain financial distress (3). Reviews in radiation oncology have similarly emphasized that financial toxicity is relevant in radiotherapy practice and that screening and supportive interventions can be integrated within routine workflows (14, 25). The access-burden pattern in the present cohort fits with a growing literature on travel burden as a cumulative temporal and financial stressor for cancer treatment, with the radiotherapy pathway being a high-risk scenario because it requires repeated attendance and may necessitate temporary relocation (20). Evidence from China on cancer-related costs likewise indicates that direct non-medical and indirect costs are important components of household burden, which can explain why travel distance, lodging needs, and cross-region care show strong descriptive relationships with financial toxicity (5, 22).

The graded relationship between financial toxicity and quality of life also mirrors prior work (4). COST-based meta-analytic evidence indicates that financial toxicity is consistently associated with worse health-related quality of life, supporting an interpretation in which financial distress contributes to multidomain impairment beyond a single “financial” domain (8, 11). The domain pattern observed in this cohort, with notable differences in global health status and emotional and social functioning, is compatible with a psycho-social mechanism in which financial strain increases worry and uncertainty, constrains engagement in supportive resources, and intensifies the perceived burden of symptoms (1, 13). At the same time, the strong concordance between COST and the QLQ-C30 financial difficulty item is expected because both capture perceived financial strain, but the correlation also supports the added value of COST as a broader measure of distress and coping, consistent with guidance from instrument custodians on the intent of COST (3, 9).

Psychological distress prevalence was high and showed a monotonic gradient across financial toxicity strata, consistent with a conceptual link between economic strain and emotional burden (4). The Distress Thermometer has been validated in Chinese cancer populations, and evidence supports a cut-off around 4 for clinically significant distress screening, which provides an appropriate basis for the present definition (5). Prior studies across settings often show that cost burden is associated with anxiety and depressive symptoms, and the present findings extend that pattern to a brief distress screening measure that is practical for clinical integration (1, 26). The persistence of an association after covariate adjustment suggests that financial toxicity captures a distress dimension not fully explained by stage, treatment modality, or performance status (27). This aligns with earlier COST validation work that reported relationships between financial toxicity and psychosocial variables while retaining distinct measurement content (9, 13).

A plausible explanatory model links the structural features of radiotherapy care with household vulnerability (3). Radiotherapy often imposes repeated appointment attendance, transportation costs, and time away from paid work, and such demands can be intensified when patients travel across regions to reach tertiary centers (2). Travel burden can also increase caregiver time commitments and lodging costs, which may be poorly protected by insurance reimbursement mechanisms (22). Several plausible pathways may link financial strain with poorer patient-reported outcomes. Financial strain may elevate uncertainty and worry and may constrain access to symptom management, nutrition support, transportation, or supportive medications (1, 26). Financial toxicity may also reduce perceived control over life circumstances, which can degrade emotional and social functioning and amplify the subjective experience of fatigue and pain (4, 13). In addition, worse clinical status can interact with financial strain by reducing capacity to work and increasing supportive care needs, producing a reinforcing cycle (14). Although cross-sectional data cannot confirm directionality, the persistence of associations after adjustment for performance status and stage supports a model in which financial toxicity operates as more than an epiphenomenon of clinical severity (8, 27).

The adjusted models also highlight an important nuance. Some access-burden variables that show strong descriptive differences across COST strata do not necessarily retain strong independent associations with distress after COST is included in the model (1). One interpretation is partial mediation: travel and lodging burdens elevate financial toxicity, and financial toxicity then captures part of the pathway to distress (2, 3). This interpretation is consistent with conceptual work framing affordability as an access dimension and positioning travel burden as a contributor to financial hardship (19, 22). From a programmatic perspective, this suggests that screening for financial toxicity may be an efficient way to identify patients bearing combined economic and access burdens, even if distance alone is an imperfect proxy (8, 20).

### Strengths, limitations, and implications

This study has several strengths. It used a multicenter design across tertiary radiotherapy settings, included a large consecutive sample, and assessed financial toxicity, quality of life, and distress using widely used patient-reported instruments with established Chinese-language applicability. The analytic approach also adjusted for multiple sociodemographic, access-burden, and clinical covariates while accounting for hospital-level structure.

Several limitations should also be acknowledged. First, the cross-sectional design precludes causal inference and does not establish temporal ordering between financial toxicity and patient-reported outcomes. Second, several measures, including out-of-pocket spending and access-burden variables, relied partly on self-report and may therefore be affected by recall bias and social desirability bias. Third, because the sample was drawn from three tertiary hospitals and excluded patients receiving exclusive palliative radiotherapy, generalizability to lower-level facilities, less intensive treatment settings, and some patients with advanced disease is limited. Fourth, patient-reported instruments are subject to measurement error, and although established measures were used, sample-specific internal consistency estimates were not re-evaluated in the final analytic dataset available for this study. Finally, although we adjusted for a broad set of covariates, residual confounding remains possible.

Clinical and policy implications follow from the consistent dose-response-like pattern across COST severity categories, with progressively worse quality-of-life and distress outcomes at lower COST levels. Routine screening for financial toxicity alongside distress screening could be implemented during radiotherapy initiation or early fractions, using brief tools and referral pathways for financial counseling, social work support, and navigation for reimbursement and assistance programs. Radiotherapy departments may reduce burden by coordinating appointment schedules, integrating supportive care services, and prioritizing patients with high travel and lodging needs for targeted help. At the policy level, findings reinforce the importance of strengthening financial protection for non-medical and indirect costs, which are prominent for radiotherapy care pathways.

### Future directions

Future research should incorporate longitudinal measurement to evaluate trajectories of financial toxicity during radiotherapy and survivorship and to clarify temporal relationships with symptom burden and distress. Intervention studies in radiotherapy settings should test pragmatic models that combine screening with navigation, financial counseling, and targeted supportive care, with outcomes that include treatment completion, symptom control, and patient-reported wellbeing. Methodologically, expanding recruitment beyond tertiary centers and incorporating objective cost data where feasible would strengthen generalizability and reduce recall bias. Finally, analytic work that separates direct medical, direct non-medical, and indirect costs can guide policy priorities, especially in settings where coverage for medical services improves faster than coverage for travel and income disruption.

## Conclusions

Among Chinese patients with gynecologic cancers receiving radiotherapy, financial toxicity is common and shows pronounced gradients by socioeconomic position, access burden, and clinical intensity. Worse financial toxicity is associated with poorer quality of life across global and functional domains, higher symptom burden, and higher psychological distress, and associations persisted after adjustment for key sociodemographic and clinical factors. The strong alignment between financial toxicity and perceived financial difficulty, together with adjusted associations with distress and functioning, suggests that financial toxicity is a clinically relevant domain in radiotherapy care. Integrating financial toxicity screening with supportive care workflows may help identify patients at elevated risk of impaired wellbeing and may inform targeted supportive responses during a period of intensive treatment demands.

## Data Availability

The raw data supporting the conclusions of this article will be made available by the authors, without undue reservation.
